# Fundamental Investigations of Bond Behaviour of High-Strength Micro Steel Fibres in Ultra-High Performance Concrete under Cyclic Tensile Loading

**DOI:** 10.3390/ma15010120

**Published:** 2021-12-24

**Authors:** Jan-Paul Lanwer, Svenja Höper, Lena Gietz, Ursula Kowalsky, Martin Empelmann, Dieter Dinkler

**Affiliations:** 1 iBMB (Institute of Building Materials, Concrete Construction and Fire Safety), Division of Concrete Construction, Faculty of Architecture, Civil Engineering and Environmental Science, Technische Universität Braunschweig, 38106 Braunschweig, Germany; massivbau@ibmb.tu-braunschweig.de; 2 ISD (Institute of Structural Analysis), Faculty of Architecture, Civil Engineering and Environmental Science, Technische Universität Braunschweig, 38106 Braunschweig, Germany; svenja.hoeper@tu-braunschweig.de (S.H.); lena.gietz@tu-braunschweig.de (L.G.); u.kowalsky@tu-braunschweig.de (U.K.); d.dinkler@tu-braunschweig.de (D.D.)

**Keywords:** fatigue, degradation, ultra-high performance fibre-reinforced concrete, tensile loading, bond behaviour, bond zone damage, damage modelling

## Abstract

The objective of the contribution is to understand the fatigue bond behaviour of brass-coated high-strength micro steel fibres embedded in ultra-high performance concrete (UHPC). The study contains experimental pullout tests with variating parameters like load amplitude, fibre orientation, and fibre-embedded length. The test results show that fibres are generally pulled out of the concrete under monotonic loading and rupture partly under cyclic tensile loading. The maximum tensile stress per fibre is approximately 1176 N/mm^2^, which is approximately one third of the fibre tensile strength (3576 N/mm^2^). The load-displacement curves under monotonic loading were transformed into a bond stress-slip relationship, which includes the effect of fibre orientation. The highest bond stress occurs for an orientation of 30° by approximately 10 N/mm^2^. Under cyclic loading, no rupture occurs for fibres with an orientation of 90° within 100,000 load changes. Established S/N-curves of 30°- and 45°-inclined fibres do not show fatigue resistance of more than 1,000,000 load cycles for each tested load amplitude. For the simulation of fibre pullout tests with three-dimensional FEM, a model was developed that describes the local debonding between micro steel fibre and the UHPC-matrix and captures the elastic and inelastic stress-deformation behaviour of the interface using plasticity theory and a damage formulation. The model for the bond zone includes transverse pressure-independent composite mechanisms, such as adhesion and micro-interlocking and transverse pressure-induced static and sliding friction. This allows one to represent the interaction of the coupled structures with the bond zone. The progressive cracking in the contact zone and associated effects on the fibre load-bearing capacity are the decisive factors concerning the failure of the bond zone. With the developed model, it is possible to make detailed statements regarding the stress-deformation state along the fibre length. The fatigue process of the fibre-matrix bond with respect to cyclic loading is presented and analysed in the paper.

## 1. Introduction

The construction industry, with its high carbon emissions, has an important role to play in progressing towards the goal of zero emissions. Maintaining the use of conventional concrete constructions and reinforced concrete materials will not reduce the emissions of carbon to stop climate change. Therefore, innovative as well as material-economical construction methods and modern high-performance materials are required. Modern ultra-high performance fibre-reinforced concrete (UHPFRC) needs twice as much cement compared to normal performance concrete, but the compressive strength increases by approximately 400%. This leads to a more favourable ratio of strength performance to carbon emissions (considering emissions from cement). After nearly 20 years of research works on UHPFRC, the current research status is notable and some countries have already launched its own specifications [1,2,3].

UHPFRC might also be suited for structures that are subjected to cyclic loading. These structures are, for example bridge, superstructures, girders of crane ways, and stands. Despite the above-mentioned specifications, clarifying the effects of cyclic loading to tensile zones of UHPFRC structures, especially the effect of the load bearing behaviour of fibres, still requires fundamental research.

Many experimental investigations deal with the bond behaviour of the reinforcement of UHPC and modelling of fatigue behaviour with respect to monotonic and cyclic loading, cf. [4,5]. In [6], pullout tests of different short- and long-hooked fibres are the basis for the modelling of bond behaviour between the UHPC-matrix and reinforcement. Experiments in [7] investigated the influence of the volume fraction and of the fibre orientation on the pullout load of reinforcement. Different analytical bond models for the description of the behaviour of the influence between reinforcement and the UHPC-matrix are represented and compared in [8]. However, fundamental investigations of the cyclic respectively fatigue behaviour of UHPFRC with respect to the bond behaviour of micro-steel fibres and its modelling are still in progress, cf. [9], where polymer fibres are taken into account.

Whereby high-strength micro-steel fibres embedded in ultra-high performance concrete (UHPC) provide a ductile failure mode under monotonic loading [9,10,11], the effect of cyclic loading might cause a rupture, resulting in an undesirable sudden failure of the structural component [12,13,14].

Fahrat [15] conducts tests on UHPFRC beams under bending with an almost fibre-free tension zone. The tests show that the fatigue strength under cyclic loading decreases considerably after only a few load cycles and is clearly below the strength under monotonic loading. Graybeal [13] investigates the flexural fatigue behaviour of uncracked UHPFRC-specimens under cyclic loading and ascertains that the fatigue strength of the concrete matrix is in the lower scatter range of the static strength. In contrast, Behloul [16] observes the fatigue behaviour of cracked UHPFRC positively influenced by steel fibres.

Abrishambaf [17], Cao [18], McSwain [19], and Wille [9] investigated more fundamentally the pullout behaviour of fibres embedded in UHPC. The test programs consist of fibre pullout tests from UHPC under monotonic loading with varying steel fibre geometries, UHPC-properties, and a number of fibres. All tests showed a continuous fibre pullout. Makita [12], Graybeal [13], Lappa [20], and Bornemann [14] also observed continuous fibre pullout, but under cyclic tensile loading. However, the observations are based on UHPFRC-specimens with a 3D fibre distribution and not on pullout tests.

Beyond that, Graybeal [13] also recognizes fibre rupture under cyclic loading during test execution based on acoustic sounds. Steel fibres are usually designed by geometry and tensile strength to provide pullout.

Makita [12] assumes that the steel fibres pull out of the concrete matrix due to cyclic loading under the top load and then slip back into the concrete channel under the bottom load by elastic rebound. Due to the cyclic pullout out and slip-back of the fibres, the fibre surface and wall of the concrete channel abrade increasingly. After a certain number of load cycles, the abrasion progresses so far that there is no longer any bond and the fibres suddenly pull out of the concrete matrix.

De Smedt et al. [21] investigate the degradation of the fibre-concrete bond by means of acoustic emissions and X-ray tomography and vary the orientation of the fibre, embedded length, and end-hook. The experiments confirm that the peak load of the bond characteristic governs the displacement rate of the fibre pullout.

To better understand the underlying processes in the microstructure, it is essential to characterize and model the load-bearing input of crack bridging fibres in tensile post-crack behaviour.

Within the tandem research project funded by the German Research Foundation in the Priority Program 2020, the different damage processes in the UHPFRC under cyclic tensile loading are fundamentally investigated. The research project contains an experimental part, which is carried out at iBMB, the Division of Concrete Construction of TU Braunschweig, and a modelling part with numerical investigations, which are performed by ISD, the Institute of Structural Analysis of TU Braunschweig. The objective of the study is to find out what happens to micro steel fibres embedded in UHPC if they are subjected to cyclic tensile loading. The results of the comprehensive test program of pullout tests should help to model the bond behaviour of steel fibres in UHPC theoretically and numerically.

## 2. Experimental Investigations

### 2.1. Pullout Tests (Groups of Fibres)

#### 2.1.1. Test Specimen, Test Program, and Test Set-Up

**Figure 1:**
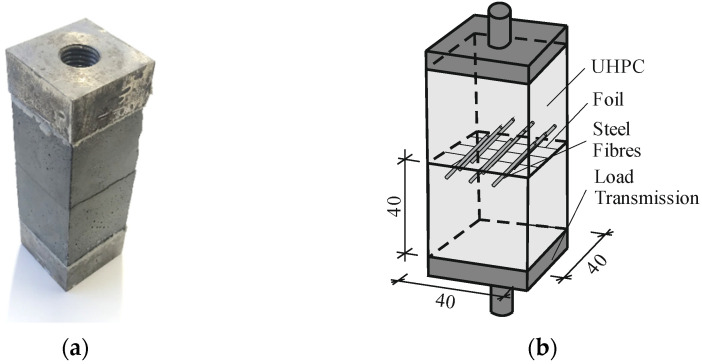
Test specimen for pullout tests: (**a**) Photo and (**b**) draft with typical dimensions in mm.

All pullout tests are performed on one type of specimen where 9 aligned fibres are placed between two UHPC-cubes, with a separating foil in between, cf. [22]. Figure 1 shows a photo and draft with typical dimensions of the test specimen.

In [23,24], the manufacturing process of the specimen is described in detail. The test program includes monotonic and cyclic loaded tests with a different length of embedment and angles of orientation. The concrete mixture is RU1 (Reference UHPC 1) [23] and had been set by the coordination of the Priority Program 2020. The steel fibre has a brass coating and tensile strength of 3575.8 N/mm^2^ as well as a measured young’s modulus of approximately 169,000 N/mm^2^, cf. [24]. Table 1 shows a brief overview of the test program.

At least three different load amplitudes for each test configuration (orientation and embedded length) are necessary to set up an S/N-curve. Considering each test configuration (orientation, embedded length, and load amplitude), the number of tests raises up to 120 including monotonic and cyclic tensile loading.

In a testing machine with a 500 N load cell, all monotonic and cyclic loaded pullout tests were conducted after at least 28 days of concrete hardening and with a load amplitude between 0.10 and 0.75 related to the maximum pullout resistance. The transducer of the testing machine measured the displacement of the test specimen. Although it might not be representative, the crack opening at the inlaid foil between the UHPC-cubes was measured with an optical laser and a camera-measuring device additionally. The comparative measurement of the crack opening confirmed the transducer’s values. However, the optical laser and camera-measuring device are not suitable to measure displacements during cyclic tensile loading.

#### 2.1.2. Test Results for Monotonic Loading

**Figure 2:**
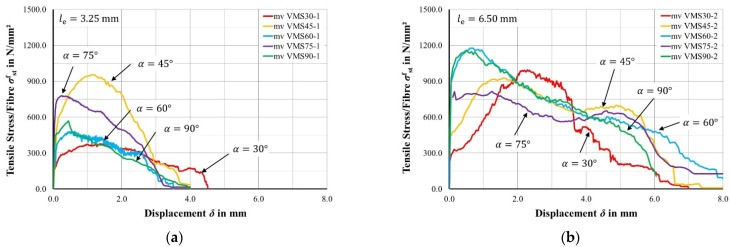
Mean load displacement curves of all tested orientations: (**a**) Embedded length of approximately 3.25 mm and (**b**) embedded length of approximately 6.50 mm.

Figure 2a,b show the tensile stress-displacement curves belonging to the embedded length of 3.25 mm (a) resp. 6.50 mm (b). The tensile stress-displacement curves are figured as mean values (mv) calculated from three individual test curves. For this purpose, a unit displacement axis with 0.02 mm steps between 0.0 and 8.0 mm is set up. For each displacement step, the relating tensile stress per fibre is searched from the three individual test curves.

The concrete age of all tests was at least 28 days and the cylinder compressive strength reached more than 150 N/mm^2^. Due to the long duration of cyclic tests, it was not always possible to start the test execution after exactly 28 days. The monotonic loaded reference tests are conducted after 28 days of hardening. The same day, the first cyclic loaded pullout test had been started with the highest load amplitude. The tests execution continued with a decreasing load amplitude. The last cyclic loaded pullout test of each test series ended approximately after 10 days after the monotonic loaded reference tests. However, bond-related hardening effects, taking place after 28 days, are negligible and do not affect the pullout behaviour significantly.

Table 2 shows the maximum pullout resistance tensile stresses related to a single fibre of the test specimen. The stresses are calculated by Equation (1):

(1) $$\sigma =F/A^{f}.$$

F is the load of the testing machine and $A^{f}$ is the diameter of the fibre with an assumed diameter of 0.19 mm (according to the manufactures specifications).

Still, there are some noticeable differences in pullout behaviour, especially regarding fibre orientation: The pullout curves with orientations of 90°, 75°, and 60°, where the fibres are mainly located in the direction of loading, show the typical load displacement behaviour like a standardized bond test, i.e., the curves rise steeply and quite quickly reach its maximum pullout resistance. Afterwards, the curves decrease almost linearly. In the rising part of the curves, 90° and 60° aligned fibres show a linear behaviour up to approximately 900 N/mm^2^, which can be attributed to the adhesion. Afterwards, the pullout resistance could be increased nonlinearly up to approximately 1200 N/mm^2^, where majorly debonding takes place, cf. also [21]. Once the fibre fully detaches from the surrounding concrete, the relative displacement rises, where friction forces mostly define the bond behaviour. Considering 75°-orientated fibres, the curves show a plateau after reaching the maximum pullout resistance, where the pullout resistance remains nearly equal. Afterwards, the curve drops quite flat at first, but then decreases very steeply.

**Figure 3:**
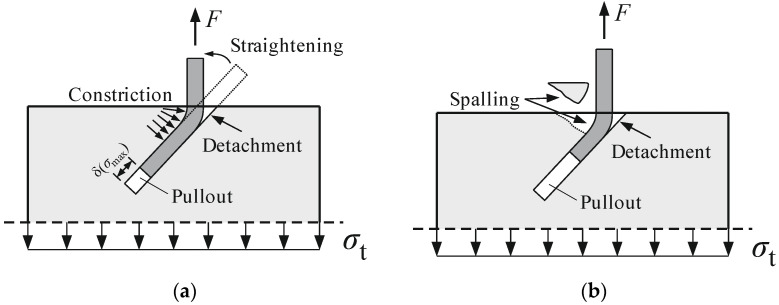
Mechanical relation and damage processes of inclined fibres with an orientation of 45° that might occur under monotonic loading: (**a**) Constriction of fibres into the concrete wall due to straightening and (**b**) spalling of concrete cones at the exit point of the fibres.

Regarding pullout tests with fibre orientations of 45° and 30°, where the axis of the fibres is mostly crossing the loading direction, a different load displacement behaviour occurs. The curves are less steep compared to 90°, 75°, and 60° and the maximum pullout resistance occurs at some relative displacement. Afterwards, the curves proceed relatively straight and drop sharply right before the fibre pulls out of the concrete. In the increasing branch of the curves, the straightening constricts the fibre into the wall of the concrete canal, see Figure 3a. High friction forces emerge between the fibre and the wall of the concrete canal. The highest constrictions resp. friction forces mark the peak point of the pullout cures in Figure 2. Afterwards, the friction forces do not increase anymore because the local tensile strength of the UHPC is exceeded. “Spalling” occurs, where little UHPC cones break out at the exit point of the fibre, see Figure 3b. From that point, the course decreases until the fibre pulls completely out of the concrete.

#### 2.1.3. Test Results for Cyclic Tensile Loading

**Figure 4:**
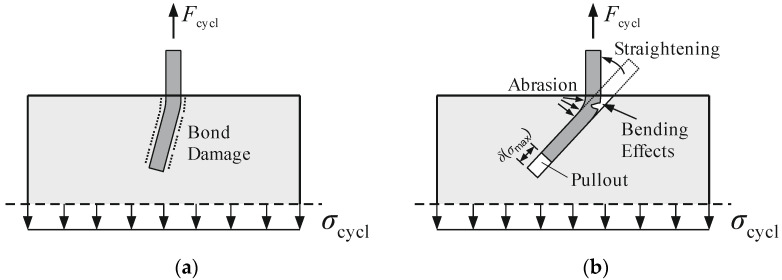
Damage processes of inclined fibres that might occur after cyclic loading: (**a**) Bond damage at an orientation of approximately 75° and (**b**) fibre damage at an orientation of approximately 45°.

The observations during the test execution showed that there are two failure mechanisms for pullout tests under cyclic tensile loading: Fibre pullout and fibre rupture [25]. These two failure mechanisms depend on the orientation of the fibres. At orientation angles of 90°, 75°, and 60°, compared to the concrete surface, the fibres were majorly pulled out of the concrete. Fibre orientation angles of 45° and 30° majorly caused fibre rupture. The damage mechanism behind that might be the intensity of friction between the fibre and concrete as well as bending effects in the fibre that come from straightening. In Figure 4a, the orientation angle is approximately 75° and there are no major damages caused by the constriction of the fibre into the concrete canal. The movement of the fibre causes damages in the bond zone and the fibre pulls out of the concrete. In Figure 4b, the orientation angle is approximately 45° and the fibre is pressed against the concrete on one side and detached from the concrete on the other side of the fibre. The combination of pressing and cyclic loading causes an abrasion and maybe also a friction fatigue of fibre material.

On the side of the fibre, where it is detached from the concrete, tensile bending effects occur increasing the applied tensile stresses. These bending effects can also lead to additional damage to the fibre’s material. The intensity of both damage effects rest upon the load amplitude and load velocity.

## 3. Theoretical Investigations

### 3.1. Bond Stress-Slip Relationship for High-Strength Micro Steel Fibres in UHPC

Already existing bond stress-slip behaviours for conventional ripped and smooth reinforcing, e.g., [26], are not applicable to high-strength micro steel fibres. The reasons for that are the different mechanical properties and different test set-ups. Furthermore, the bond stress-slip behaviours (for conventional bar reinforcing) are only valid for orthogonal pullout, whereas the fibre distribution in UHPFRC is undirected. Therefore, new bond models are required to describe the pullout behaviour of high-strength steel fibres embedded in UHPC.

First, the measured loads of the executed pullout tests need to be converted into a bond stress. Equation (2) gives the formulae to calculate the bond stress:

(2) $$\tau_{st}^{f}=\frac{F_{st}^{f}}{\pi \cdot \emptyset^{f}\cdot \left(l_{e}-\delta \right)}.$$

Since the embedded length decreases during the fibre pullout, Equation (2) contains the term $\left(l_{e}-\delta \right)$. In that, parameter $l_{e}$ is the maximum embedded length and δ is the measured displacement (of the measuring devices). Furthermore, $F_{st}^{f}$ is the measured load and $\emptyset^{f}$ is the diameter of the fibre, assumed to be 0.19 mm [24].

The measured displacement δ is assumed to be the slip *s* of the fibre tip, although there is an elastic strain of the fibre. However, a calculation of the elastic strain is very small [27] and can be disregarded.

**Figure 5:**
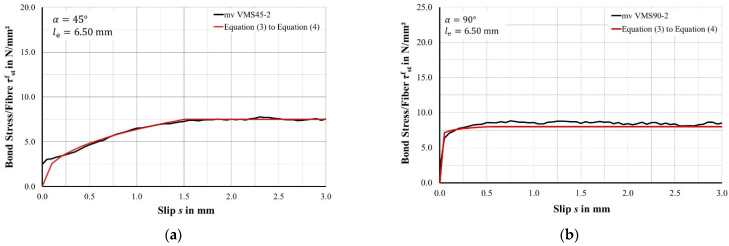
Experimental bond stress-slip relationship and analytical bond stress-slip relationship according to Equations (3) and (4): (**a**) Fibre orientation angle at 45° and (**b**) fibre orientation angle at 90°.

Figure 5 shows the calculated bond stress-slip relationship for different fibre orientations and embedded lengths, exemplary for 45° and 90°, see Table 1.

The major distinctions rest upon the various orientations. A high inclination of fibres in pullout tests, e.g., 45°, show in the branches of the curves that the pullout resistance rises steadily within a slip of 1.5 mm. From that point, the curve proceeds horizontally and the bond stress remains constant. This means the full performance of fibres with an orientation of e.g., 45° requires a slip of 1.5 mm or, related to UHPFRC parts, 1.5 mm of crack width. Crack widths like that are not applicable in construction due to durability reasons. That is why inclined fibres might never reveal their full performance.

In contrast to that, pullout tests with higher fibre inclinations, e.g., 90°, reach a maximum of pullout resistance earlier than 1.5 mm and proceed horizontally afterwards. The maximum pullout resistance occurs at different points in the branches of the curves depending on the orientation of the fibre. Generally speaking, the lower the inclination of the fibre, the higher the relative displacement between the concrete and fibre in reaching maximum pullout resistance. Pullout tests with an orientation of 45° reach its ultimate pullout resistance at a relative displacement of approximately 1.5 mm, 60°-inclined fibres at 1.0 mm, 75°-inclined fibres at 0.75 mm, and 90°-inclines fibres at 0.50 mm. The relation of peak pullout resistance and relative displacement rests upon the bond stiffness caused by a combination of constriction and bending effects of the fibre, see Section 2.1.2.

The various bond stiffnesses that depend on the various orientations of the fibres should be expressed by a general analytical bond stress-slip behaviour. Since some of the pullout curves of steel fibres resemble the pullout curves of conventional reinforcement, the formulary of the existing bond stress-slip behaviour, cf. [26], might also be used to set up a bond stress slip relationship for steel fibres. However, the parameters of the formulary must be adjusted to the bond behaviour of steel fibres. The first part of the analytical bond stress-slip behaviour, Equation (3), represents the rising part of the bond curve:

(3) $$\tau_{st}^{f}=\tau_{max}^{f}\cdot {\left(\frac{s}{s_{1}}\right)}^{\kappa_{1}}\;\mathrm{for}\;0<s\leq s_{1}.\;$$

$\tau_{max}^{f}$ stands for the ultimate bond stress and $\kappa_{1}$ is a parameter that describes the bond stiffness. Parameter s is the slip of the fibre and parameter $s_{1}$ marks the relative displacement, at which point the maximum bond strength occurs. After reaching the ultimate bond strength, the analytical bond stress-slip relationship proceeds horizontal at the level of the ultimate bond strength $\tau_{max}^{f}$. Equation (4) shows the formulae for the horizontal course:

(4) $$\tau_{st}^{f}=\tau_{max}^{f}\;\mathrm{for}\;s_{1}<s\leq l_{e}.$$

The curve of Equation (4) proceeds horizontally until the fibre completely pulls out of the concrete. The pullout occurs when the slip s equals the embedded length $l_{e}$. As already mentioned earlier, parameters $s_{1}$ and $\kappa_{1}$ depend on the orientation of the fibres. Table 3 lists its values for each fibre orientation between 30° and 90°.

Table 3 shows a correlation between the orientation angle α of the fibres and parameters $s_{1}$ and $\kappa_{1}$ of the analytical bond stress-slip relationship. The test program contains five fibre orientations in steps of 15° between 30° and 90° to gather the most possible information to the bond behaviour of inclined steel fibres. It is not possible to test all fibre orientations but since there is a correlation between the bond curves and orientation angle, it is also not necessary. The missing orientations between those 15° steps are considered by an interpolation. The best results came from an exponential regression between both coefficients $s_{1}$ and $\kappa_{1}$ and the angle of orientation α shown in Equations (5) and (6):

(5) $$s_{1}=2.4\cdot \exp\left(-0.042\cdot \alpha \right)$$

(6) $$\kappa_{1}=4.1\cdot \exp(-0.023\cdot \alpha ).$$

Equations (5) and (6) are inserted in Equation (3) giving the general equation for the analytical bond stress slip relationship depending on the slip *s* and angle of orientation α:

(7) $$\tau_{st}^{f}=\tau_{max}^{f}\cdot {\left(\frac{s}{2.4\cdot \exp\left(-0.042\cdot \alpha \right)}\right)}^{4.1\cdot \exp(-0.023\cdot \alpha )}.\;$$

The approach in Equation (7) gives the opportunity to describe the degradation of bond behaviour depending on the orientation of an embedded high-strength micro steel fibre in UHPC. The approach gives the opportunity to evaluate the fibre bearing effect in an UHPFRC-component at different stages of slips, respectively crack widths.

### 3.2. S/N-Relationship for Fibre Pullout

**Figure 6:**
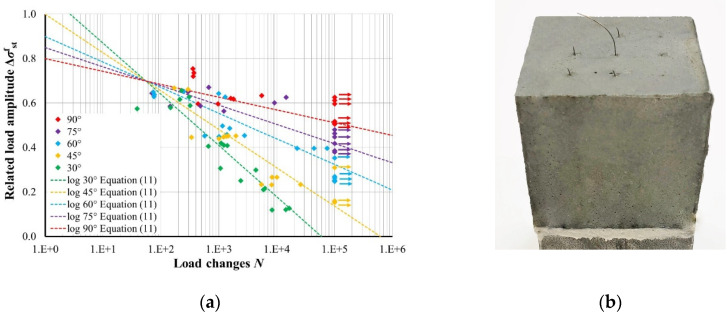
Results of cyclic tensile loaded pullout test: (**a**) S/N-diagram of pullout tests with different orientations and (**b**) photo of a test specimen after cyclic loading with ruptured and pulled-out fibres.

The stress amplitude and load cycles from the cyclic loaded pullout test of Table 1 have been plotted into an S/N-diagram with a semi-logarithmic depiction. Test specimens that did not fail before 100,000 load cycles are marked as run-outs shown by an arrow pointing to the right. Figure 6a shows the plotted S/N-diagram and regression lines.

The S/N-diagram shows that there is a significant deterioration of the fatigue resistance depending on the orientation of steel fibres. The combination of constricting the fibre into the wall of the fibre channel on the one side of the fibre and emerging bending effects on the other side cause major damage, e.g., abrasion, to the fibres material and leading to fibre rupture, see Figure 6b. The degradation of the embedded fibre is higher at lower orientation angles, which can also be reflected from the inclination of the S/N-lines in Figure 6a.

It might also be concluded from Figure 6a that there is a centre of rotation of the individual S/N-curves of each orientation. The centre of rotation is located at a load amplitude of 0.7 and approximately 80 load cycles. From that point, the S/N-curves decrease linearly with different slopes. The equation for each S/N-curve regarding the orientation are given in Equation (8) as a logarithmic function:

(8) $$\Delta \sigma_{st}^{f}=A\cdot \ln N+B.$$

Two parameters *A* and *B* are variables in Equation (8) that define the slope of the S/N-curve, i.e., the fatigue degradation. The higher the fatigue degradation, the higher the slope of the S/N-curve.

The degradation parameters *A* and *B* depend on the orientation α of the fibres. This relationship is reflected in Equations (9) and (10):

(9) A=−0.07·ln[α]+0.34

(10) B=−0.28·ln[α]+2.05.

Both degradation parameters are inserted into Equation (8) leading to the following equation:

(11) $$\Delta \sigma_{st}^{f}=\left(0.07\cdot \ln\left[\alpha \right]-0.34\right)\cdot \ln N-\left(0.28\cdot \ln\left[\alpha \right]-2.05\right).\;$$

Equation (11) gives the opportunity to describe the fatigue behaviour of embedded steel fibres in UHPFRC considering the load amplitude, number of load changes, and fibre orientation α. Equation (11) is only valid for the UHPC and fibre type used in this study. The extension of Equation (11) to other concrete and fibre material requires tailored experimental and theoretical investigations.

Even though fibre rupture occurred during the test execution only at some orientations, under certain circumstances, this can also cause a sudden failure of a UHPFRC component and needs to be investigated more in detail. One starting point could be the choice of the fibre. So far, the fibres in these research project have a slenderness of $l_{f}/\emptyset_{f}=13.0/0.19=68$. In order to avoid fibre rupture under cyclic tensile loading in any case, a lower slenderness should be chosen for the same fibre material. However, further pullout tests with different fibre geometries and tensile strengths are required to determine an optimal fibre design that is also suitable for cyclic tensile loading.

## 4. Numerical Investigations

Experiments dealing with fibre pullout phenomena usually give an overall load-displacement characteristic. A deeper insight into the phenomena of the bond degradation along the fibre is hardly possible, since it would need very complex CT equipment, acoustic emissions, or X-ray tomography, cf. [21], which had to be applied during the test. Nonetheless, detailed information on the kind and size of degradation could be the basis for further optimization of the fibre geometry and surface.

3D Finite Element analyses of the discrete fibre-matrix composite structure are performed to investigate the relation between the local bond strength and pullout behaviour of a single fibre from the matrix in order to transfer the findings to the modelling of the load-bearing behaviour of the composite UHPFRC. The calibration of the following presented bond model is based on experimental fibre pullout tests under monotonic tensile loading [11,23,28] and is comprehensively explained in [23,27].

### 4.1. Geometric Model to Describe the Fibre-Matrix Composite

**Figure 7:**
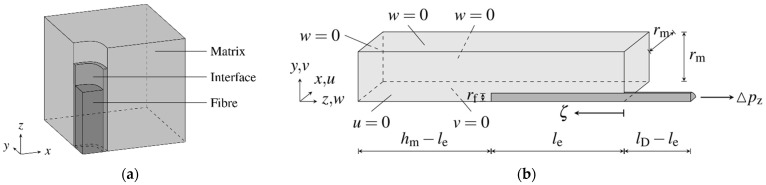
Geometric model of fibre-matrix composite (adapted from [27]): (**a**) General scheme and (**b**) Finite-element-model for simulation of a pullout test with geometry parameters and boundary conditions.

The numerical analysis of the fibre-matrix composite requires a discrete description of the geometries of fibre and concrete matrix in order to simulate the relative displacement of the components and to provide information about the three-dimensional composite mechanisms and composite stress distribution along the fibre. The developed geometric bond model is shown schematically in Figure 7a. The bond zone is modelled by isoparametric zero-thickness interface elements based on the derivation according to [29]. The extension by a node-mapping algorithm generates a connectivity matrix assigning the opposite contact surfaces of the active displacement situation, cf. [27,30]. A contact condition concerning the normal direction enables the description of a possible loss of contact, a limit value defines the tolerance of the mapping in tangential direction. Together with the material model of the bond zone, a rigid as well as a sliding bond can be described.

Since the analysis is performed by means of the Finite Element Method, the geometry of the model can be extended to different orientations of the fibre with respect to the cracked surface, length, diameter, hooks, and waviness, if necessary.

### 4.2. Material Model of Fibre-Matrix Bond Zone

**Figure 8:**
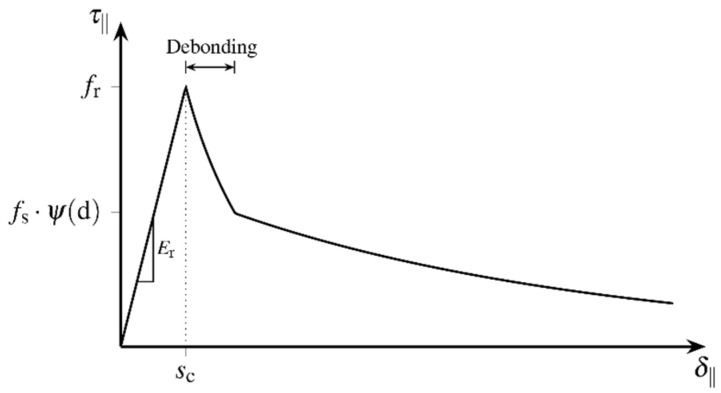
Schematic representation of the bond behaviour with increasing relative displacement regarding the developed bond model (reprinted from [27]).

The presented bond zone model describes the development of the local bond behaviour between fibre and matrix as a function of the active relative displacement δ adapted to the model equations from [31] and takes into account the damage-induced degradation of the bond capacity, cf. [27]. Figure 8 schematically illustrates the course of the bond stress related to the relative displacement regarding pure shear loading of the bond zone.

The bond behaviour of the different phases is described assuming an additive decomposition into rates of elastic and inelastic relative displacements:

(12) $$\overset{\cdot }{\delta }={\overset{\cdot }{\delta }}^{el}+{\overset{\cdot }{\delta }}^{in},$$

cf. [32]. Simplifying, the rigid bond is modelled here employing elastic relative displacements $\delta^{el}.$ The sliding bond is described by irreversible relative displacements $\delta^{in}$. The irreversible local debonding characterizes the transition between the phases. The linear relation between the relative displacement $\delta^{el}$ and the bond stress τ is given by:

(13) $$\tau =E_{r}\cdot \delta^{el}.$$

The three-dimensional stress state at the bond zone contains shear stresses $\tau_{\|}$ and normal stresses $\tau_{⊥}$ acting parallelly and normally to the contact surface, respectively. Thus, relative displacements $\delta_{\|}$ parallel and $\delta_{⊥}$ normal to the contact surface are to be distinguished. The strength of the bond zone is defined as bond capacity $\tau_{lim}\left(d\right)$ related to the bond zone damage d, which is formulated as a function of the irreversible relative displacements $\;\delta^{in}$ [32]. The bond zone damage d takes into account the bond mechanisms adhesion and micro-interlocking that are independent from lateral pressure. Lateral pressure increases static as well as sliding friction.

**Figure 9:**
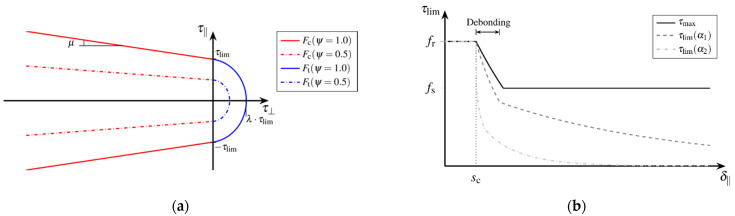
Failure model (reprinted from [27]): (**a**) Failure surface addicted to the degradation ψ(d) and (**b**) evolution of bond capacity with increasing relative displacement.

Reaching the bond capacity is defined by two complementary failure criteria. Concerning lateral compression this yields:

(14) $$F_{c}=\left|\tau_{\|}\right|+\mu \left(d\right)\cdot \tau_{⊥}-\tau_{lim}\left(d\right)\leq 0$$

with the coefficient of friction μ. Regarding lateral tension the deviation of the bond tensile strength from the shear strength is accounted for by the anisotropy coefficient λ. As long as there is contact between the surfaces, the failure criterion:

(15) $$F_{t}=\tau_{\|}^{2}-\mu \left(d\right)\cdot {\left(\tau_{lim}\left(d\right)-\tau_{⊥}\right)}^{2}+\left(1-\mu \left(d\right)\right)\cdot \left({\left(\frac{\tau_{⊥}}{\lambda }\right)}^{2}-\tau_{lim}^{2}\left(d\right)\right)\leq 0$$

is to be satisfied both when the contact surfaces are detaching from each other and when sliding bond behaviour arises. Figure 9a represents the failure surface of the multisurface model according to the degradation:

(16) ψ(d)=1−d.

The non-associated slip rules define the rates of the inelastic relative displacements as functions of the normal bond stresses to:

(17) $${\overset{\cdot }{\delta }}_{c}{}^{in}=\overset{\cdot }{\lambda_{c}}\cdot \frac{\partial Q_{c}}{\partial \tau }\;\mathrm{and}\;{\overset{\cdot }{\delta }}_{t}{}^{in}=\overset{\cdot }{\lambda_{c}}\cdot \frac{\partial Q_{t}}{\partial \tau }.\;$$

The potential functions Q are described as a function of the coefficient of lateral-pressure evolution η(d):

(18) $$Q_{c}=\left|\tau_{\|}\right|+\eta \left(d\right)\cdot \tau_{⊥}-\tau_{lim}\left(d\right)\;\mathrm{and}\;$$

(19) $$Q_{t}=\tau_{\|}^{2}-\eta \left(d\right)\cdot {\left(\tau_{lim}\left(d\right)-\tau_{⊥}\right)}^{2}+\left(1-\eta \left(d\right)\right)\cdot \left({\left(\frac{\tau_{⊥}}{\lambda }\right)}^{2}-\tau_{lim}^{2}\left(d\right)\right).$$

Therefore, the bond capacity $\tau_{lim}$ is defined in accordance with the different bond phases and takes into account the degradation ψ of the bond zone. The process of debonding presumes increasing damage. Unloading of the bond zone cannot restore the initial rigid bond behaviour due to adhesion, if debonding of the contact surfaces has started. Thus, the irreversible change in bond capacity is modelled as a function of damage. Figure 9b schematically represents a possible evolution of the bond capacity $\tau_{lim}$ when varying the model parameter α, that scales the damage growth and therefore implicitly takes into account the effects of degradation, see Equation (22).

The maximum bond capacity $\tau_{max}$ summarizes the bond mechanisms adhesion and micro-interlocking that act independent from lateral pressure, in terms of a bond strength to:

(20) $$\tau_{max}\left(d\right)=\{\begin{array}{c} f_{r}\;\\ f_{r}-\left(f_{r}-f_{s}\right)\cdot (d/d_{d})\;\\ f_{s} \end{array}\begin{array}{c} \mathrm{for}\;\\ \;\mathrm{for}\;\;\\ \mathrm{for}\; \end{array}\begin{array}{c} d\;\leq \;0\\ 0\leq d\leq d_{d}\\ d_{d}\leq d \end{array}\begin{array}{c} \mathrm{rigid}\;\mathrm{bond}\\ \;\mathrm{local}\;\mathrm{debonding}\\ \;\;\;\;\mathrm{sliding}\;\mathrm{bond}. \end{array}$$

The bond capacity $\tau_{lim}\left(d\right)$, the coefficient of friction μ(d), and the coefficient of lateral-pressure evolution η(d) with respect to damage growth are defined as functions of the degradation ψ(d) to:

(21) $$\tau_{lim}\left(d\right)=\tau_{lim}\left(d\right)\cdot \psi \left(d\right),\;\mu \left(d\right)=\mu_{max}\left(d\right)\cdot \psi \left(d\right)\;\mathrm{and}\;\eta \left(d\right)=\eta_{max}\left(d\right)\cdot \psi \left(d\right).\;$$

The increase of the bond zone damage d is given with the rate of the inelastic relative displacement $\;{\overset{\cdot }{\delta }}_{\|}{}^{in}$ and the fibre circumferential length $u_{f}$ as:

(22) $$\;\overset{\cdot }{d}\;=\beta \left(d\right)\cdot \frac{\left|\;{\overset{\cdot }{\delta }}_{\|}{}^{in}\right|}{u_{f}}\;\mathrm{with}\;\beta \left(d\right)=\alpha \cdot \left(1-d\right),\;\alpha >0.$$

Thereby the potential of the damage evolution β(d) determines the influence of the rate of relative displacements to the damage increase. While at the beginning of the sliding bond, the existing micro aggregate interlock is sheared off and thus a significant fraction of the bond resistance is destroyed, for finely ground particles further abrasion of contact surfaces causes only a small increase in damage corresponding to the model conception. The constant scaling factor α describes the bond quality dependent of damage evolution, see Figure 9b.

The bond model presented here describes the local bond behaviour phenomenologically regarding rigid and sliding bond states as well as the main bond mechanisms, cf. [27]. Therefore, it is predestined to describe the fatigue behaviour in the bond zone. Thereby, bond fatigue due to frequently recurring loading and unloading of the bond zone corresponds to progressive degradation. In accordance with elasto-plastic modelling, low stresses located inside the failure surface, see Figure 9a, have no effect on the bond capacity. Only the local exceeding of the initial bond strength initiates the evolution of bond zone damage and therefore, a degradation of the bond capacity.

### 4.3. Numerical Analyses Concerning the Fatigue Behaviour of the Bond Zone

The fatigue of the bond zone is characterized by damage-induced structural loosening as well as progressive detachment of the contact surfaces from each other. For fibre-reinforced UHPC, the weakening of the bond zone due to cyclic loading means a significant reduction in resistance and load-bearing capacity. The effects of cyclic tensile loading on a partially-detached fibre embedded in UHPC are investigated in the following numerical analyses. The geometry and the boundary condition of the simulated fibre pullout are shown in Figure 7b and the associated parameters are given in Table 4. For more information concerning the model calibration cf. [23,27].

**Figure 10:**
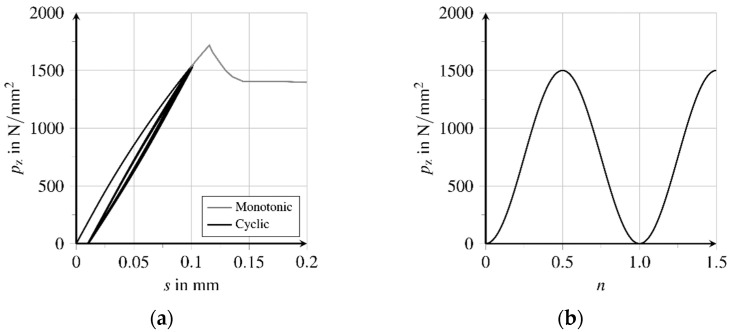
Simulation of cyclic tensile loading (adapted from [27]): (**a**) Loading over slip relation and (**b**) sinusoidal tensile cyclic loading function.

**Figure 11:**
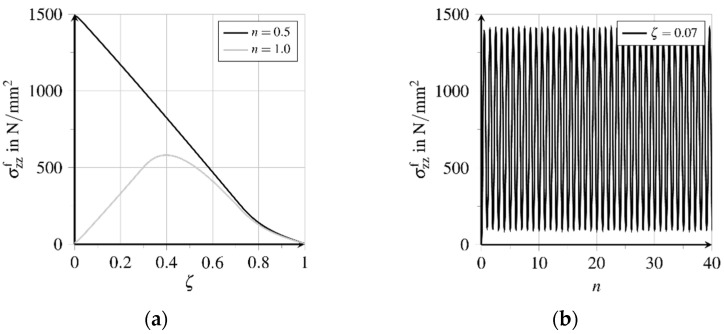
Evolution of longitudinal fibre stresses (adapted from [27]): (**a**) Stress distribution over bond length in first load cycle and (**b**) stress evolution near the fibre outlet at further load cycles.

The stress range of the cyclic tensile loading is chosen with $\Delta p_{z}=$1500 N/mm^2^. This is below the load-bearing capacity of the fibre-matrix composite structure under monotonically-increasing loading, which has been identified to be slightly above 1700 N/mm^2^, see Figure 10a. Thus, during initial loading, a part of the fibre is detached from the matrix. Changes in the stress-deformation state in the boundary layer can be found within a few loading cycles. The sinusoidal cyclic tensile loading is provided at the fibre tip, whereby n represents the number of load cycles, see Figure 10b. For the evaluation of the fatigue processes in the bond zone, at first, the state of the bond zone after the first load change is of interest. With initial loading of the fibre up to the maximum stress of $\Delta p_{z}=$ 1500 N/mm^2^, a partial detachment of the fibre from the matrix can be stated. At unloading the fibre, the bond resistance in the detached area prevents complete retraction of the relaxing fibre, so that a residual stress state remains in the fibre-matrix composite, see Figure 11a.

Figure 11a shows the distribution of longitudinal stresses $\sigma_{zz}^{f}$ along the embedded fibre, expressed with the related bond length ζ, see Figure 7b, at the first loading and unloading, i.e., in the first load cycle. The comparison of the stress courses demonstrates that the composite structure is not relieved along the second half of the embedded fibre with ζ→1.0, while the tensile stress disapperas at the top of the composite. Figure 11b depicts the stress range just below the top of the composite at ζ=0.07, which shows no significant change in the fibre stress state after only a few load cycles.

**Figure 12:**
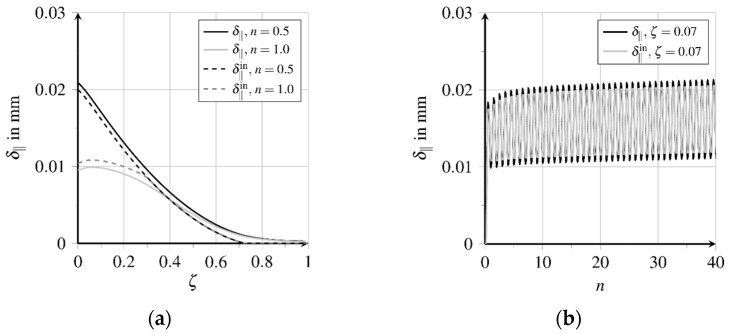
Evolution of relative displacements (adapted from [27]): (**a**) Displacement distribution over bond length in first load cycle and (**b**) displacement evolution near the fibre outlet at further load cycles.

**Figure 13:**
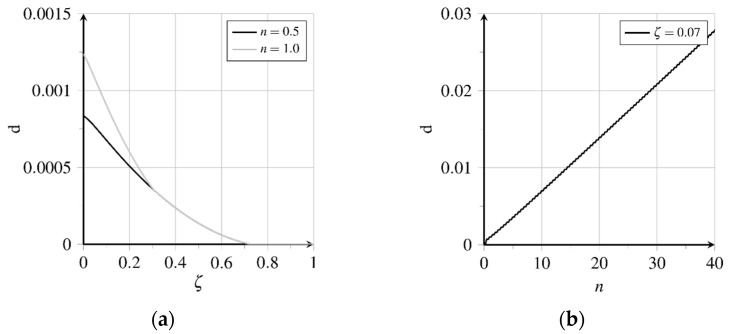
Evolution of bond zone damage (adapted from [27]): (**a**) Damage distribution over bond length in first load cycle and (**b**) damage evolution near the fibre outlet at further load cycles.

**Figure 14:**
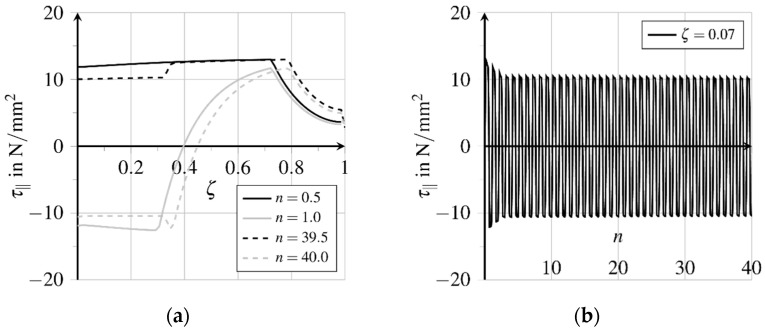
Evolution of bond zone shear stresses (adapted from [27]): (**a**) Stress distribution over bond length in first load cycle and (**b**) stress evolution near the fibre outlet at further load cycles.

Due to pullout loading, longitudinal and transverse strains develop proportional to longitudinal and transverse stresses, which cause relative displacements between the fibre and matrix. These relative displacements, generated in the bond layer at loading, are partially irreversible. With the irreversible transition from rigid to sliding bond behaviour inelastic relative displacements as well as bond zone damage are generated along about 70.0% of the bond length, see Figure 12a and Figure 13a. The thereby activated bond mechanisms sliding friction and micro-interlocking lead to an almost constantly distributed high bond resistance, see Figure 14a. Figure 12a depicts the relative displacements $\delta_{\|}$ acting parallelly to the contact surface at first loading and unloading. The evolution of the relative displacements at position ζ=0.07 for further load cycles is shown in Figure 12b. The inelastic part follows from the detachment of the fibre from the matrix, called debonding. Unloading the fibre, the total relative displacement is reduced up to about ζ=0.7, while the inelastic part is reduced only at the first 30.0% of the bond length. At further load cycles, the relative displacement near the fibre outlet increases continuously.

Figure 13a shows the bond zone damage with three different areas concerning the state of the bond zone. Regarding the embedded end of the fibre rigid bond without damage of the bond zone prevails. Between about ζ=0.7 and ζ=0.3, no significant increase of damage can be expected because unloading generates no decrease of the inelastic relative displacements, as the remaining bond resistance prevents the fibre from relaxing, see Figure 12a. In contrast, the bond zone damage will increase near the fibre outlet due to further movements both at the loading and unloading of the fibre, see Figure 13b.

Bond zone damage increases almost linearly here, see Figure 13b. Due to the chosen formulation of the damage evolution potential β(d), see Equation (22), and a constant rate of the inelastic relative displacement flattening of the course of bond zone damage evolution can be expected at higher numbers of load cycles. Because of the change of the movement direction, both the matrix and fibre surface are polished and smoothened, cf. [33]. Weakening the bond zone results in fatigue and finally, failure with progressive fibre pullout, cf. [33]. The comparison of the distributions of the bond shear stresses $\tau_{\|}$ indicates a shifting of the different bond zone areas regarding maximum loading and unloading times of the first and 40th load cycle respectively, see Figure 14a. The displacement courses presented in Figure 12a show a sign change of the course of elastic relative displacements and the related bond shear stresses at ζ=0.4, see Figure 14a. The bond zone experiences a reversed shear loading from the fibre outlet to ζ=0.4, which induces a reversed inelastic relative displacement evolution at simultaneously increasing bond zone damage d, see Figure 13b. Simultaneously, the absolute amount of inelastic slip is reduced in this area.

**Figure 15:**
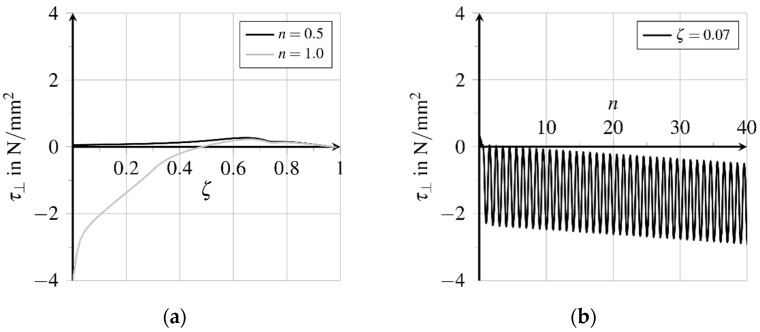
Evolution of bond zone normal stresses (adapted from [27]): (**a**) Stress distribution over bond length in first load cycle and (**b**) stress evolution near the fibre outlet at further load cycles.

Due to the increasing bond zone damage, the bearable bond shear stress is reduced. Thereby cracking in the contact zone progresses and, at the same time, the fatigue-relevant area extends from the fibre outlet to the middle of the bond zone. Figure 15a illustrates the distribution of the bond normal stresses regarding the bond length. At unloading, the vanishing lateral contraction of the fibre generates a contact pressure at the region of the fibre outlet because of the inelastic expansion of the bond zone, which is not reduced by unloading the fibre matrix composite structure.

**Figure 16:**
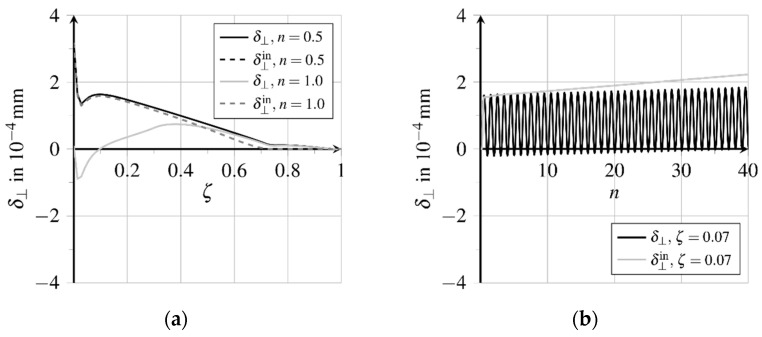
Evolution of normal relative displacements (adapted from [27]): (**a**) Displacement distribution over bond length in first load cycle an (**b**) displacement evolution near the fibre outlet at further load cycles.

Figure 16a,b illustrate the relative displacements perpendicular to the bond zone for both total and inelastic displacements, respectively. The inelastic part increases with each load cycle, see Figure 16b, while the elastic part directed in the opposite direction generates a lateral compressive stress. The increasing difference between the inelastic and total relative displacement near the fibre outlet explains the significant increase of the lateral pressure due to friction regarding further load cycles, see Figure 15b. The enhancement of the sliding friction counteracts the degeneration of the bond capacity due to increasing bond zone damage. Therefore, the amplitude of the cycling shear stress in the bond zone remains almost constant near the outlet of the fibre, see Figure 14b.

The decreasing bond strength near the outlet of the fibre enforces a progressive debonding of the fibre from the matrix regarding the end of the fibre due to equilibrium between fibre pullout force and bond shear stress. This encloses the enlargement of the range of influence of the inelastic relative displacements at the outlet of the fibre, cf. [34]. Finally, the complete debonding of the fibre initiates the failure of the bond zone not being able to bear the load any longer. Both a sudden pullout of the fibre from the matrix and a gradual fibre pullout due to high contact pressure near the outlet of the fibre are possible failure modes. For more information on the debonding process cf. [27,34,35].

Cracking in the contact zone that progresses due to cyclic loading of the fibre-matrix composite structure acts as the governing driver of bond zone failure because it initiates the irreversible decrement of bond resistance, cf. [34]. The proposed bond zone model does not include any enforcement of the damage evolution due to lateral compression in the bond zone. However, in the model there exists an interaction between contact pressure and bond zone resistance, which in turn regulates the magnitude of the inelastic relative displacements. Accordingly, an increased lateral compression in the bond zone may slow down bond zone fatigue. While high external lateral compression is unlikely regarding a tensile stressed cross section, a high displacement-induced evolution of lateral compression at fibre pullout may significantly enlarge the failure resistance of the bond zone by retarding cracking in the contact zone and reducing the magnitude of inelastic relative displacements after contact surfaces have detached from each other. Fatigue of the bond zone particularly affects the load-bearing capacity of cracked UHPFRC, since damage-inducing relative displacements may occur between the contact surfaces with the partial detachment of the fibre from the matrix, cf. [27].

The numerical investigations of the fibre pullout with respect to cyclic loading gives deep insight into the phenomena during loading and unloading conditions. By means of the detailed information on the degradation process, optimization of the fibre geometry and properties with respect to the debonding along the fibre is possible.

## 5. Conclusions

The interaction of the experimental test programme and the numerical investigations provides useful and fundamental insights into the fatigue and degradation behaviour of the fibre-matrix composite for further research work. The test programme comprises 120 pullout tests with varying parameters, such as fibre-embedding length, fibre orientation, and load amplitude, from which theoretical modelling approaches are derived. From experimental tests, no information about the stress distribution along the fibre can be obtained. To better understand the phenomenological processes of fibre-matrix bond behaviour, a mechanism-oriented bonding model was developed for three-dimensional FE analyses. The following conclusions can be derived from this paper:

1. Under monotonic loading continuous pullout always occurred, despite the orientation angle of the fibre. The test results of the cyclic loaded pullout tests show that cyclic loading can lead to fibre rupture.
2. An analytical bond stress-slip relationship set up for monotonic loading depends on the angle of fibre orientation.
3. The S/N-curve of the pullout tests under cyclic loading shows a correlation between the fatigue resistance respectively to the fatigue degradation and the angle of fibre orientation. Fatigue degradation is higher with a decreasing orientation angle.
4. At tensile cyclic loading, the fatigue of the bond resistance starts at the crack surface and propagate during load cycles till the end of fibres. Due to inelastic deformation, residual stresses occur and may influence bond capacity.
5. The investigations show that the bond mechanisms adhesion and micro aggregate interlock are decisively influencing the maximum pullout resistance. The maximum pullout resistance is only reached with the beginning of microcrack opening in the matrix, when the detachment of the fibre from the matrix is completed.

The obtained results create the basis for the scale transition to describe the load-bearing behaviour of UHPFRC at a macro level. On the experimental side, the analytical bond stress-slip relationship for high-strength micro steel fibres can be aligned to every single fibre in a UHPFRC-component. Converting the bond stress-slip relationship into a tensile stress or a force in the fibre and summing them up and distributing them evenly over the components cross section, leads to a post-cracking tensile strength of the UHPFRC. The same process could be used for cyclic tensile loading where, instead of the analytical bond stress-slip behaviour, the appropriate S/N-curve is aligned to the fibres. The stresses could also be summed up to calculate fatigue stress for UHPFRC-components.

Simulations of large-scale constructions require a homogenization strategy that describes the coupled phenomenological processes of the interacting material components as well as the random anisotropic fibre orientation. The represented numerical investigations are fundamental to model the composite behaviour of UHPFRC under cyclic tensile loading on the macro level.

## Figures and Tables

**Table 1:** Test program of pullout tests.

Series	Orientation [°]	Embedded Length ^2^ [mm]	Load Amplitude	Amount
VMS	30/45/60/75/90	6.50/3.25	-	30
VMZ			0.10–0.75 ^1^	90

^1^ load amplitudes vary due to the electromechanical drive of the testing machine. ^2^ may vary due to inaccuracies in manufacturing.

**Table 2:** Maximum pullout resistance stress in N/mm^2^ according to Figure 2.

Series	Embedded Length	Embedded Length	*F*_3.25_/*F*_6.50_
	3.25 mm	6.50 mm	-
VMS30	376.9 ± 41.2	993.6 ± 66.6	0.38
VMS45	955.3 ± 135.8	953.6 ± 151.9	1.00
VMS60	480.7 ± 71.0	1176.3 ± 26.7	0.41
VMS75	778.8 ± 52.0	884.0 ± 77.7	0.88
VMS90	568.7 ± 42.4	1159.1 ± 53.3	0.49

**Table 3:** Parameters for analytical bond stress-slip relationship.

Series	Orientation α	$\tau_{\max}^{f}\;[N/{\mathrm{mm}}^{2}]$	$s_{1}\;[\mathrm{mm}]$	$\kappa_{1}$
VMS30	30	10.0	2.00	0.60
VMS45	45	7.5	1.50	0.40
VMS60	60	8.0	1.00	0.20
VMS75	75	6.0	0.75	0.10
VMS90	90	8.0	0.50	0.05

**Table 4:** Geometrical and material parameters for simulation of pullout test (data from [27]).

Parameter	Symbol	Unit	Value
Fibre diameter ^1^	$d_{f}$	mm	0.19
Wire length ^1^	$l_{D}$	mm	16.5
Embedding fibre length ^1^	$l_{e}$	mm	6.5
Matrix radius ^1^	rm	mm	2.0
Matrix height ^1^	hm	mm	40.0
Initial Young’s modulus of bond zone	$E_{r}^{\;}$ ^2^	N/mm^2^	13,000.0
Bond strength (rigid bond)	$f_{r}$	N/mm^2^	13.0
Bond strength (sliding bond)	$f_{s}$	N/mm^2^	10.3
Coefficient of anisotropy	λ	-	1.0
Damage due to debonding	$d_{d}$	-	0.002
Coefficient of friction	μ	-	0.15
Coefficient of lateral pressure evolution	η	-	0.001
Damage evolution coefficient	α	-	0.025

^1^ see Figure 7b. ^2^ begin of sliding bond/elastic limit $s_{c}$ = 0.001 mm.
